# Identification of the Conformational transition pathway in PIP_2_ Opening Kir Channels

**DOI:** 10.1038/srep11289

**Published:** 2015-06-11

**Authors:** Junwei Li, Shouqin Lü, Yuzhi Liu, Chunli Pang, Yafei Chen, Suhua Zhang, Hui Yu, Mian Long, Hailin Zhang, Diomedes E. Logothetis, Yong Zhan, Hailong An

**Affiliations:** 1 Key Laboratory of Molecular Biophysics, Hebei Province, Institute of Biophysics, School of Sciences, Hebei University of Technology, Tianjin 300401, China; 2 Center of Biomechanics and Bioengineering and Key Laboratory of Microgravity (National Microgravity Laboratory), Institute of Mechanics, Chinese Academy of Sciences, Beijing 100190, China; 3 School of Electrical and Electronics Engineering, Shijiazhuang Tiedao University, Shijiazhuang 050043, China; 4 Key Laboratory of Neural and Vascular Biology, Ministry of Education, The Key Laboratory of Pharmacology and Toxicology for New Drug, Hebei Province, Department of Pharmacology, Hebei Medical University, Shijiazhuang 050017, China; 5 Department of Physiology and Biophysics, School of Medicine, Virginia Commonwealth University, Richmond, Virginia 23298, US

## Abstract

The gating of Kir channels depends critically on phosphatidylinositol 4,5-bisphosphate (PIP_2_), but the detailed mechanism by which PIP_2_ regulates Kir channels remains obscure. Here, we performed a series of Targeted molecular dynamics simulations on the full-length Kir2.1 channel and, for the first time, were able to achieve the transition from the closed to the open state. Our data show that with the upward motion of the cytoplasmic domain (CTD) the structure of the C-Linker changes from a loop to a helix. The twisting of the C-linker triggers the rotation of the CTD, which induces a small downward movement of the CTD and an upward motion of the slide helix toward the membrane that pulls the inner helix gate open. At the same time, the rotation of the CTD breaks the interaction between the CD- and G-loops thus releasing the G-loop. The G-loop then bounces away from the CD-loop, which leads to the opening of the G-loop gate and the full opening of the pore. We identified a series of interaction networks, between the N-terminus, CD loop, C linker and G loop one by one, which exquisitely regulates the global conformational changes during the opening of Kir channels by PIP_2_.

Inwardly rectifying potassium (Kir) channels allow greater K^+^ influx at membrane voltages more negative rather than efflux at voltages more positive than the potassium equilibrium potential. Kir channels are involved in a wide range of physiological processes, such as maintaining stable resting membrane potentials, controlling cell excitability, shaping the initial depolarization, regulating cardiac rhythm, vascular tone, insulin release, and salt flow across epithelia^1^,^2^,^3^, as well as the final repolarization of action potentials in many cell types, including heart cells^4^,^5^,^6^,^7^,^8^.

There are 16 members in the Kir family belonging to seven subfamilies (Kir1-7). Kir channels are homo- or hetero- tetramers of four subunits. Each subunit has a simple transmembrane domain (TMD), containing an outer transmembrane helix (M1), an ion-selective P loop (selectivity filter - SF), and an inner transmembrane helix (M2). Besides the M1-P-M2 motif, there exist a cytoplasmic domain (CTD), which is composed of N- and C- termini located on the intracellular side of the membrane forming a large ‘cytoplasmic pore’ structure. The C-linker, containing a dozen amino acids, connects the TMD and CTD^9^,^10^. The extension of the transmembrane permeation pathway to the cytoplasm is one unique characteristic feature of Kir family members^11^.

Kir channels are regulated by several different cellular factors, such as G-proteins, ATP, pH, some of which act specifically on specific subfamily members (nucleotides-Kir6, G-protein-Kir3, intracellular Na^+^-Kir3.2, 3.4, and extracellular K^+^ and Mg^2+^-Kir2)^12^,^13^,^14^. The anionic lipid phosphatidylinositol 4,5-bisphosphate (PI(4,5)P_2_, known as PIP_2_) on the other hand is needed to activate all members of eukaryotic Kir channels^13^. Kir channel regulation is accomplished by a conformational change that allows the protein to switch between two alternative (closed vs. open) conformations, a process known as gating. The cytosolic domain is highly conserved among the Kir subfamilies and forms the cytosolic vestibule^15^,^16^,^17^, which, together with the transmembrane pore, generates a long ion conduction pore^18^. There are three gates along the long central pore of Kir channels: the selectivity filter gate, the inner helix gate (both in the transmembrane pore) and the G-loop gate (in the cytoplasmic pore)^13^. Functional studies and mutagenesis data have suggested that positively charged residues in the N- and C-termini determine sensitivity of Kir channels to PIP_2_ activation^19^,^20^,^21^,^22^,^23^,^24^,^25^,^26^. These positively charged residues of the C-linker are capable of forming electrostatic interactions with the negatively charged head group of PIP_2_. Recently, atomic structures of Kir2.2^10^ and Kir3.2 bound to PIP_2_^27^ have been solved identifying residues that bind PIP_2_ directly. On the basis of static crystallographic structures, two possible gating models have been described as follows. The first model named “twist model”, which was proposed by Bavro and colleagues, was based on the structure of KirBac3.1 S129R (PDB code: 3ZRS)^9^. The hypothesis is that the rotation of the cytoplasmic domain (CTD) prepares the C-linker and brings the slide helix into register with the CD loop to open the inner helix gate^9^. The second model, named “upward motion model”, was proposed by Hansen and colleagues based on the structure of cKir2.2 (PDB code: 3SPI)^10^. This model suggested that an upward motion of the CTD translates towards and becomes tethered to the transmembrane domain (TMD), the G-loop first inserts into the TMD and then opens the inner helix gate^10^. However, the detailed gating mechanism of Kir channels induced by PIP_2_ remains obscure.

Among Kir channels, Kir2.1 channel is activated by PIP_2_ alone, serving as an excellent model with which to understand the detailed gating mechanism induced by PIP_2_. Here, we have combined molecular dynamics (MD) with targeted MD simulations to address the conformational transition pathway in the gating of the Kir2.1 channel. Our data show that Kir2.1 channel gating unfolds in a step-by-step process as follows. First, with the upward motion of the CTD, the C-linker forms a new helix and the Kir2.1 channel achieves an “activated” state but the pore remains in the closed state. Second, the kink of the C-linker triggers the rotation of the CTD which pulls the pore open. Moreover, we identified a series of interaction networks that controls the PIP_2_-induced conformational changes during Kir channel gating. Based on the data, a brand new gating model is proposed which will shed light on understanding the molecular mechanism of PIP_2_ gating of Kir channels.

## Methods

### Homology Modeling

Homology models of the full-length mouse Kir2.1 channel were performed using the SWISS-MODEL server^28^,^29^,^30^. The target sequences were taken from Genbank (http://www.ncbi.nlm.nih.gov/Genbank/). The template structures were the chicken Kir2.2 (PDB code: 3JYC^31^ and 3SPI), and the mouse Kir3.2 (PDB code: 3SYQ). These homology models were based on chain A of the template structures, in which the two missing loops, one that connects the N-terminal beta-strand and the slide helix, and one that connects the two transmembrane alpha-helices, were completed. All the models were evaluated with QMEAN (The QMEAN4 score is a composite score consisting of a linear combination of 4 statistical potential terms (estimated model reliability between 0–1)^32^. The pseudo-energies of the contributing terms are given their Z-scores with respect to scores obtained for high-resolution experimental structures of similar size solved by X-ray crystallography. The members of the inwardly rectifying K^+^ channel family exhibit high degree of sequence similarity. Due to high sequence homology (about 78%, 77% and 53% sequence identity to chicken Kir2.2 and mouse Kir3.2 respectively), the QMEAN4 score was 0.79, 0.566 and 0.545 respectively and QMEAN Z-score was −4.54, −3.36 and −3.66 respectively. Each model was compared to its template to verify that the modeling step had not significantly altered backbone and side chain conformation.

### Conventional Molecular Dynamics

For the full-length Kir2.1 channels simulation, the channels were immersed in an explicit palmitoyloleoyl-phosphatidylcholine (POPC) bilayer generated by the VMD membrane package^33^. The whole system was then solvated, and K^+^ and Cl^−^ of ~150 mM were positioned randomly among the solvent to neutralize the system. We built three systems (Closed, Activated, and Open states). Each system involved ~140,000 atoms in the MD simulation. Full three-dimensional periodic boundary conditions were used. Each system was in a rectangular box with the size of 101 × 98 × 156 Å^3^ (Closed state), 101 × 99 × 156 Å^3^ (Activated state), and 104 × 105 × 153 Å^3^ (Open state), respectively. The solvated systems then underwent four equilibration steps: (i) 4 ns of equilibration with melting of lipid tails, (ii) the entire protein was fixed for 5 ns, enabling reorganization of the lipid and solution (iii) 5-ns extensive equilibration with protein released, (iv) finally letting the system relax freely for over 10–20 ns until reaching equilibrium. Each Targeted Molecular Dynamic is performed on the equilibrated system.

ALL MD simulations were performed in with the NAMD2 program (http://www.ks.uiuc.edu/Research/namd/)^34^ and the CHARMM 27 force filed^35^. A scaling factor for 1–4 interactions of 1.0 was applied in all simulations. Langevin dynamics and the Langevin piston were used to maintain the temperature at 310 K and a pressure of 1 atm with a damping coefficient of 1 ps–1. The barostat oscillation and damping time scale for the Langevin piston method^36^,^37^ were set to 200 fs and 50 fs, respectively. The van der Waals interactions were modeled using the Lennard-Jones potential. Short-range interactions was smoothed at 10 Å and truncated at 12 Å. Long-range electrostatic forces were taken into account using the Particle Mesh Ewald (PME) method (120 × 120 × 180 grid points)^38^,^39^. Non-bonded and PME calculations were performed on every time step. The time step employed was 2 fs, and pair lists were updated every 20 iterations. The coordinates were saved every 4 ps for analysis. Pore dimensions were evaluated using HOLE^40^. For molecular visualization and structural diagrams we used VMD. Simulations were carried out on a 64-processor Linux cluster.

### Targeted Molecular Dynamics (Targeted MD)

In Targeted MD^41^, a subset of atoms in the simulation is guided towards a final ‘target’ structure by means of steering forces. The force on each atom is given by the gradient of the potential:


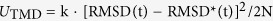


where k is the force constant, RMSD(t) is the instantaneous best-fit RMSD of the current coordinates to the target coordinates, RMSD*(t) is the preset RMSD value for the current time step, and N is the number of targeted atoms. The number of atoms used to calculate the RMSD from the target structure was set to be the same as the number of restrained atoms. The targeted RMSD value was decreased monotonically from the initial RMSD to the target structure until it reached near 0 Å at the end of the Targeted MD simulation. The Targeted MD method generates the trajectory of the conformational transition by using an external force between the starting and the target molecular conformation^42^,^43^,^44^,^45^,^46^.

## Results

### Constructing 3D structural models in different conformational states of Kir2.1

**Figure 1:**
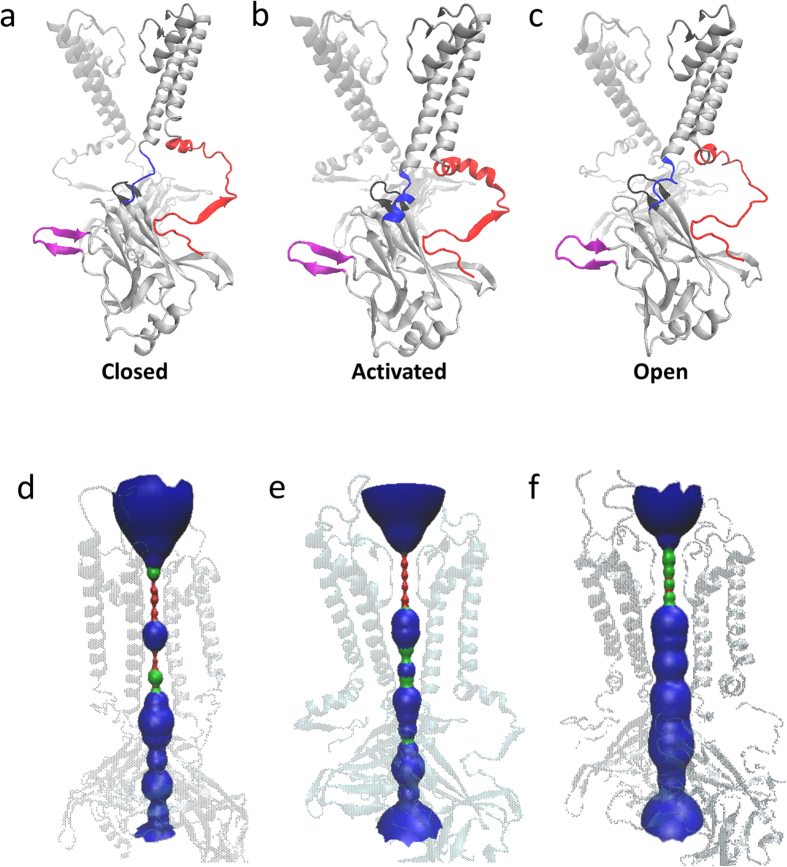
Schematic diagrams of the structures of Kir2.1 in closed (**a**), activated (**b**) and open (**c**) states. C-linker, N-terminal and G-loop are highlighted as blue, red and black, respectively. (**d**–**f**) are the pore lining of closed, activated and open channel, respectively. The pore lining generated using HOLE is shown as a red (radius < size of water), green (radius ≈ size of water) and blue (radius > size of water). All the structures are generated using VMD.

**Figure 2:**
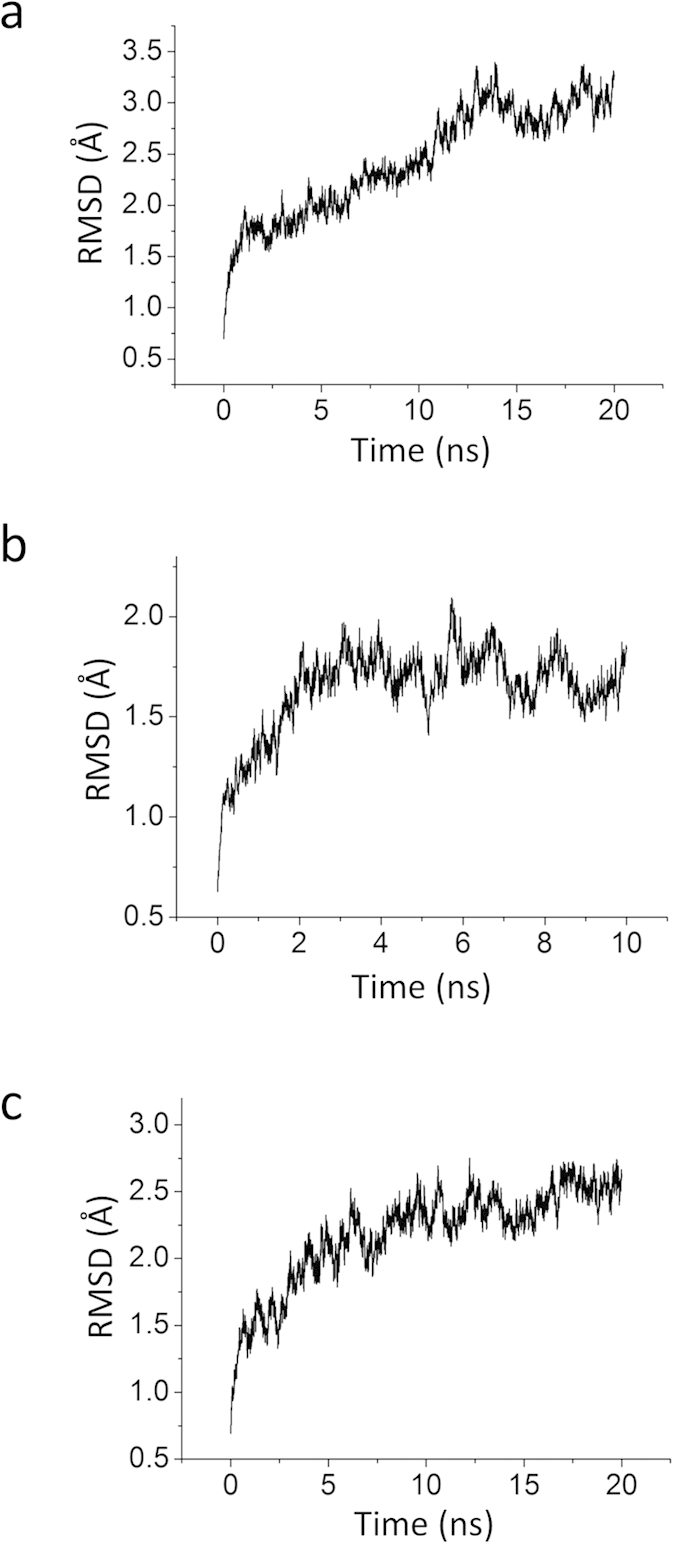
RMSD variations for three systems (**a**, Closed. **b**, Activated. **c**, Open.) throughout the simulation. RMSDs were calculated based on all the Cα atoms of the channel.

As shown in Fig. 1a–c, we constructed three 3-dimentional structures of Kir2.1 channels. The crystal structure of chicken Kir2.2 and mouse Kir3.2 with PDB ID 3JYC^31^, 3SPI and 3SYQ are chosen as the templates. The sequences identities are 78%, 77% and 53%, respectively. Based on the radius of the pore (Fig. 1d–f), we named them closed, activated and open states of Kir2.1, respectively. During 10–20 ns free MD simulations, these three structures reach their equilibration states whose RMSD values are 3 Å or less (Fig. 2). Comparing the three structures, we identified the main conformational difference which is the C-linker. Electrophysiological experiments and crystallographic data show that there are PIP_2_ binding sites at the C-linker.

Next, we proceeded to perform a series of Targeted MD simulations on the C-linker to confirm the conformational pathway involved in the gating of Kir channels.

### Formation of a helix of the C-linker induces a 5 Å upward motion of the CTD pushing the pore to the activated state

**Figure 3:**
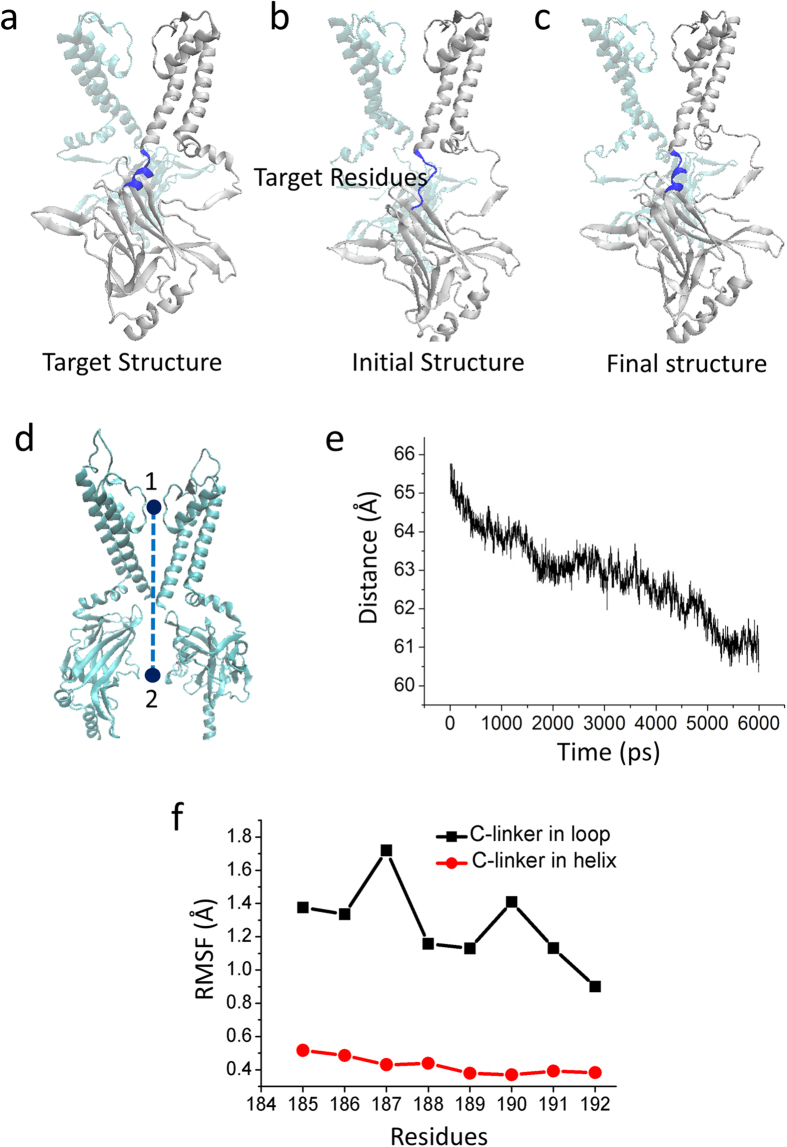
The CTD transfers 5 Å towards the TMD during the first Targeted MD simulation which focuses on the C-linker (blue). (**a**) The schematic diagram of the targeted structure, which is in the activation state. (**b**) The schematic diagram of the initial structure, which is in the closed state. The target residues, to which the targeted force is applied (residues Lys185-Thr192), are colored blue. (**c**) The schematic diagram of the final conformation, which is achieved by the Targeted MD simulation. (**d**) Definition of the distance (R) which is from the center of the selectivity filter (the upper sphere) to the center of the C-terminal domain (CTD) (the lower sphere). (**e**) Evolution of the distance R vs. time. (**f**) RMSFs of the residues in C-linker, when the conformation of the C-linker is in the helical (black) and in the loop states (red).

To test whether the conformational change of the C linker could open the channel, we first performed Targeted MD simulation at the C-Linker of Kir2.1 which are highlighted in blue in Fig. 3a–c. The C-Linker at the helical state in the activated Kir2.1 channel was set as the target structure (Fig. 3a, the blue helix). During the Targeted MD simulation, an external force was applied to the backbone atoms of the C-linker residues (Lys185 to Thr192) with a force constant of 500 kcal/mol/Å^2^, which pushed the C-Linker to change from a loop to a helix. After 6 ns Targeted MD, the final structure of the C-Linker changed from loop to helix (Fig. 3c). During this process, the distance R (the R is the distance from the center of the selectivity filter to the center of the CTD) decreased from 65.6 Å to 60.8 Å, showing about 5 Å upward movement of the CTD toward the membrane (Fig. 3d, e). The RMSF (root mean squared fluctuation) shows that the transition from a loop to a helical structure produced a more rigid C-linker (Fig. 3f), which enhanced the connection between the TMD and CTD. Thus the pore reached the activated state.

### The rotation of the CTD pulls the pore to the open state

**Figure 4:**
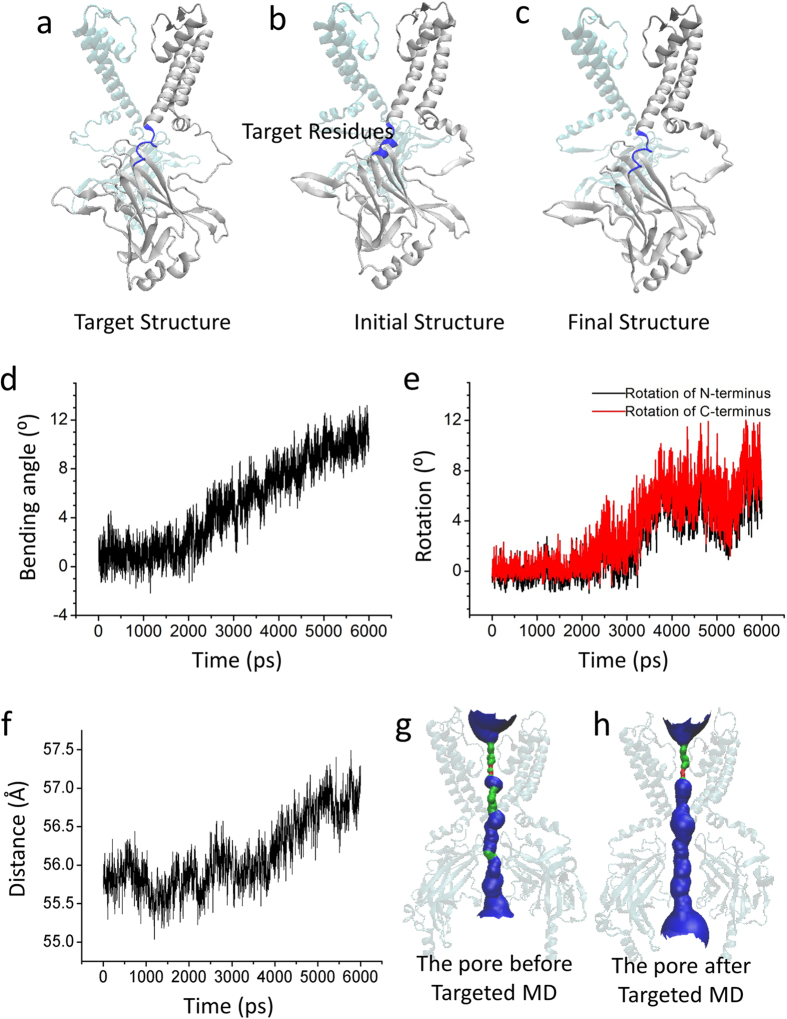
The rotation of CTD pulls the channel to the open state. (**a**) The schematic diagram of targeted structure, which is in the open state. (**b**) The schematic diagram of the initial structure, which is in the activated state. The target residues, to which the targeted force is applied (residues Lys185-Thr192 and PIP_2_ binding sites), are colored blue. (**c**) The schematic diagram of the final conformation, which is achieved by our last Targeted MD simulation. (**d**) The time course of the kinking of the C-linker. (**e**) The time course of the rotation of the N- (black) and C-termini (red). There is an 8° rotation-angle of the CTD the during Targeted MD simulation. (**f**) The distance, R, vs. the simulation time. (**g**) and (**h**) show the schematic pore lining by Kir2.1 (generated using HOLE) for the initial-activated state and the final conformations achieved by the simulations, respectively. The pore lining generated using HOLE is shown as a red (radius < size of water), green (radius ≈ size of water) and blue (radius > size of water).

We next performed a Targeted MD simulation, which started with the activated state targeting the structure at the open state (Fig. 4a, b). The final structure is shown in Fig. 4c. During this simulation, an external force was applied to the backbone atoms of the C-linker (Lys185 to Thr192) (blue) and the PIP_2_ binding sites (Arg80, Trp81, Arg82, Lys182, Lys185, Pro186, Lys187, Lys188, Arg189 and Arg218) with a force constant of 500 kcal/mol/Å^2^, which made the C-linker start to bend at 2 ns, by up to about 12° (Fig. 4d). At the same time, the N/C-termini also started to rotate at 2.5 ns, by up to about 8° around the channel axis in an anticlockwise direction (viewed from the membrane) (Fig. 4e)^47^. The C-linker transitioned from a helical to a semi-helical state (Fig. 4c). The CTD showed a small downward movement at 3.6 ns (Fig. 4f) with the pore achieving the full open state (Fig. 4g, h).

### The twisting of the C-linker induces a kink of the M2 which opens the inner helix gate

**Figure 5:**
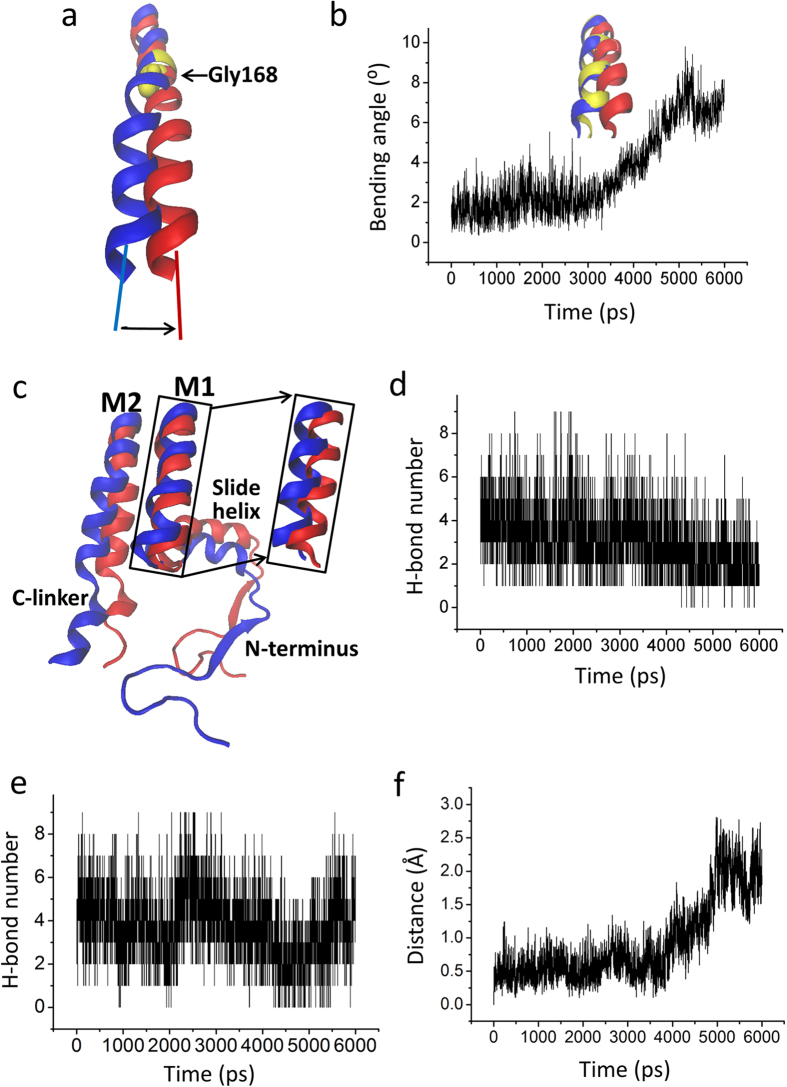
Bending of inner M2 helix opens the bundle-crossing gate. (**a**) The kink of M2 helix at the conserved glycine hinge (Gly 168) from the closed state (blue) to the conformation (red). (**b**) Evolution of the bending angle of M2 vs. the simulation time. Inside panel shows the conformation of the inner M2 helix at 0 ns (blue), 3 ns (yellow), and 6 ns (red) during the Targeted MD simulation. (**c**) Partial structure in the final Targeted MD conformation (red) compared to the initial conformation (blue). (**d**) and (**e**) are the time courses of the H-bond forming between outer M1 and inner M2 helix, and between slide helix and M1 helix, respectively. (**f**) The time courses of the distance of the center of slide helix upward movement to the membrane.

More generally, the mechanistic details of opening of the inner helix and G-loop gates are far from clear. Compared to the activated state, the lower part of the M2 bends at a highly conserved glycine, the hinge residue^48^,^49^,^50^, whereas the structure above the conserved glycine remains relatively rigid. The M2 helix starts to bend at approximately 3.6 ns, by up to about 8° (Fig. 5a, b)^9^,^51^. As shown in Fig. 4a–c, the C-linker connects the inner helix M2 and the CTD. The C-linker in a helical state shows high rigidity, which strengthens the connection between M2 and the CTD (Fig. 3f). Our data show that there are two parts that contribute to bending the M2 helix. First, the bending of the C-linker contributes to the kink of the M2 helix (Figs 4d and 5c). The upward motion of the slide helix starting at 3.6 ns facilitates the bending of the M2 helix (Fig. 5c, f). Second, the Interactions between the M1 and M2 helices and between the slide helix and between M1 helix contribute to a conformation change of M1 making the M2 helix bend (Fig. 5d, e).

### The G-loop bounces away from the CD loop, which opens the G-loop gate

**Figure 6:**
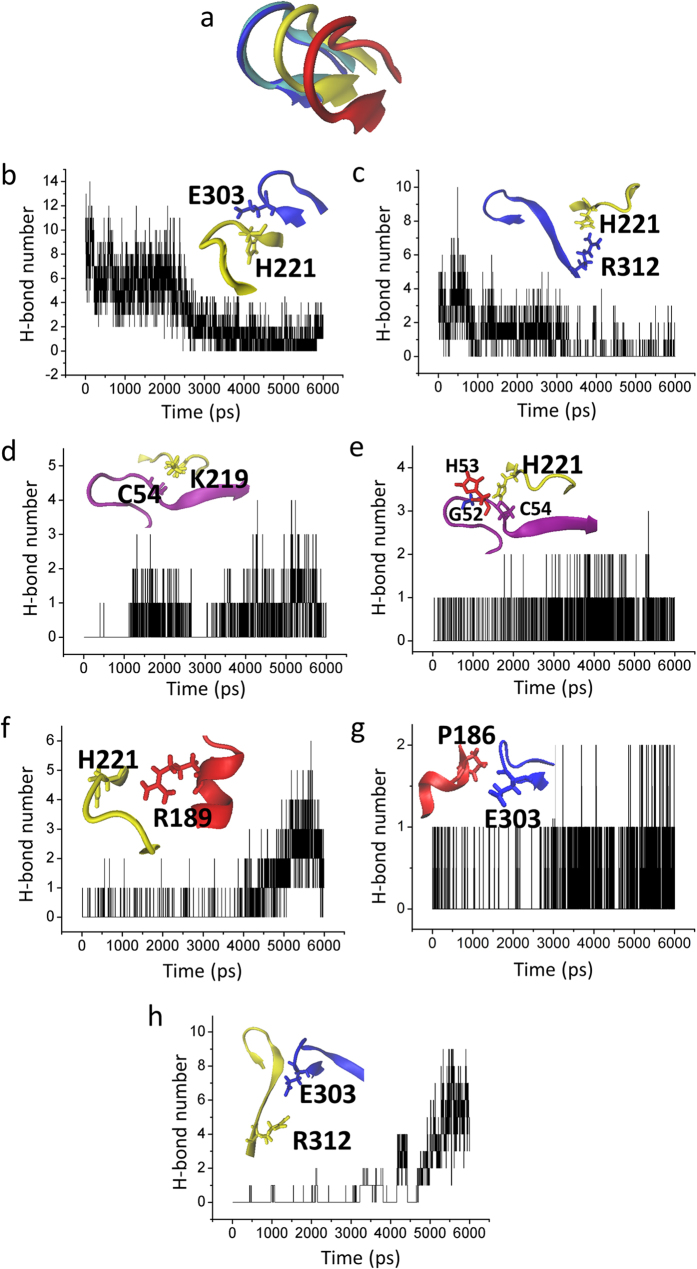
The interaction network that opens the G-loop gate. (**a**) The distinct conformations of G loop at 0 ns (blue), 2 ns (silver), 3 ns (yellow), and 6 ns (red) during the Targeted MD simulation. (**b**) and (**c**) show the time course of the interaction between G-loop (blue) and CD-loop (yellow) through intrasubunit E303-H221 (**b**) and intersubunit R312–H221 (**c**). (**d**) and (**e**) show the time course of interactions between N-terminus (purple) and CD-loop (yellow) through C54–K219 (**d**) and G52/H53/C54-H221 (**e**). (**f**–**h**) show the strengthened interactions between CD-loop (yellow) and C-linker (red) through H221–R189 (**f**), between C-linker (red) and G-loop (blue) through P186–E303 (**g**) and between adjacent G-loops through E303–R312 (**h**).

Besides the inner helix gate located near the inner leaflet of the bilayer, the G-loop gate is located at the CTD along the pore. As shown in Fig. 6a, the G-loop moves significantly as the channel achieves its open state. In this Targeted MD simulation, we identified three interaction-networks. The interactions between G loop and CD loop (E303-H221 and R312-H221) are weakened (Fig. 6b, c), which destabilizes the closed state of the G-loop gate. The intrasubunit interactions between the N-terminus and the CD loop are strengthened, namely C54 with K219 and G52, H53, C54 with H221 (Fig. 6d, e) which enhances the connections between the N terminus and the C terminus. The intersubunit interactions between the CD loop and the C-linker (H221-R189), between the C-linker and the G loop (P186-E303) and between the adjacent G loops (E303-R312) are strengthened (Fig. 6f–h), which enhances the connections between the C terminal, C-linker and G loop. We speculate that these interactions between N-terminus, CD loop, C linker and G loop are gradually strengthened, which will stabilize the G loop in the open state.

### Gating model of Kir channels

The data obtained in this study now allow us to propose a more detailed model of PIP_2_-induced gating of Kir channels. The first conformational change of the CTD is a 5 Å upward translocation towards the TMD which is pushed by the C-linker to change from a loop to a helical structure. The pore of the Kir channel is still closed. But the kink of the C linker, which is involved in interactions with PIP_2_ triggers the rotation of the CTD (Fig. 4d, e). The rotation of the CTD causes the C-linker to partially uncoil because of the same direction of the rotation of the CTD and the uncoiling of C-linker (Figs 4e and 5c). The C-linker switches from a helix to a semi-helix, which engages the further rotation of the N/C-terminus (Fig. 4e) which triggers a small downward movement of the CTD (Fig. 4f) and the upward movement of the slide helix (Fig. 5f)^52^,^53^. The two movements cause the kink of the M2 helix (Fig. 5b), ultimately opening the inner helix gate (Fig. 5h)^9^,^51^. The rotational movement or twist of the CTD could therefore conceivably promote opening of the inner helix gate^16^,^47^,^54^. With rotation of the N-terminus, the interaction between G loop and CD loop is broken and then the bouncing away of the G loop leads to the opening of the G loop gate (Fig. 6a–c). The strengthened interaction between N-terminus, CD loop, C-linker and G loop not only stabilize the G loop gate in the open state (Fig. 6d–h), but also can stabilize the inner helix gate (Fig. 6f, g). The gating model we propose here is similar to previous gating models^52^,^53^,^55^, but our current model provides greater details than the former ones leading to channel full opening.

## Discussion

PIP_2_-induced gating of Kir channel is an intrinsically dynamic process and difficult to understand by the few available static crystal structures that are missing the key transition steps connecting them. There have been various studies aiming to understand the molecular mechanism of Kir channel gating^51^,^56^,^57^,^58^,^59^,^60^,^61^,^62^. However, since allosteric conformational changes, such as the PIP_2_-driven gating of Kir channels, take place on the microsecond time scale, it is not possible to capture the transfer of a closed channel to an open one even through 100 ns-long MD^56^ or 500 ns-long Coarse-Grained MD^58^ simulations. To accomplish the transition from the closed to the open state of full-length Kir channels, we performed Targeted MD simulations, which was developed by Schlitter *et al.*^41^,^63^ and has been used to study a variety of allosteric transitions in large proteins^42^,^43^,^44^,^45^,^46^,^64^,^65^,^66^,^67^.

Here we applied Targeted MD to study the gating of Kir2 channels. We have focused on the gating changes induced by the C-linker (a domain at the C-terminus of the inner helix). We performed a two step-targeted MD on a modeled mouse full-length Kir2.1 channel. To the best of our knowledge, this is the first time Targeted MD simulations have been utilized aiming to understand the detailed gating mechanism of Kir channels.

Comparing available crystal structures of Kir channels, we noticed that the C-linker can change conformation from a loop to a helix in a PIP_2_-dependent manner. If PIP_2_ is replaced by PPA, which contains as a head group only phosphoric acid, the head group does not interact with the CTD and cannot maintain the helical structure of the C-linker. This suggests that to keep the C-linker in a helical structure PIP_2_ is needed^10^. This is compatible with a recent functional study showing that small head group anionic lipids failed to activate Kir channels in the absence of PIP_2_^68^. PIP_2_ facilitated the cytoplasmic domain movement toward the membrane. A vertical motion of the Kir domains seems to be a critical step for channel opening^13^,^69^,^70^. So the C-linker changes conformation from a loop to a helix, which is the first step toward Kir channel pore opening. The best-characterized lipid modulator of Kir channel activity is the anionic membrane phospholipid, PIP_2_, only 1% of the acidic lipid in the whole cell^71^,^72^,^73^. PIP_2_ mainly binds to the N-terminal end of the slide helix, at the domain connecting the TMD and CTD (C-linker)^10^,^22^,^74^,^75^. The channel-PIP_2_ interaction providing a tangential force may facilitate the bending of the C-linker which causes the rotation of the CTD, and then the C-linker uncoils. Limited by the methods, there is no PIP_2_ in our Targeted MD simulations. During the simulation, we applied force on the PIP_2_ binding residues and hoped that the applied forces, in somehow, mimic the channel-PIP_2_ interactions. Following this idea, we propose that the same direction of the uncoiling of C-linker and the rotation of the CTD causes a further counterclockwise rotation of CTD (Fig. 4e) to trigger a small downward movement of CTD (Fig. 4f) and an upward movement of the slide helix (Fig. 5f) driving the kinking of the M2 helix (Fig. 5b) to open the inner helix gate. The secondary anionic phospholipids including PG, PA, PS, PI, and CL can potentiate the high PIP_2_ sensitivity^68^. The synergistic PL(-) can pull the N-terminal Slide Helix towards the membrane to increase the PIP_2_ affinity. Our results are consistent with the conformational effect, which can stabilize the open state^47^. The Slide Helix is important for channel gating. For example, Slide Helix mutations were reported to cause Barter’s syndrome (in Kir1.1)^76^,^77^, neonatal diabetes (in Kir6.2)^78^ and Andersen-Tawil (in Kir2.1)^79^.

Following our previous notion^57^ which was that the interaction networks between the N termini, CD-G loops and C-linker play a critical role in the PIP_2_-induced gating of Kir channels, we focused on the interactions between these residues. Our data show that the rotation of CTD of Kir2.1 alters interactions between the G-loop and CD-loop, thus breaking interactions between H221 of the CD-loop and the tip (H221-E303) (Fig. 6b) or the base of the G-loop (H221-R312) (Fig. 6c). Liberation of the two residues allowed the G-loop to “bounce back” and open wide enough for K^+^ ion passage. Three new interactions are then formed, namely E303 interacts both with intersubunit P186 and R312, H221 interacts both with R189 and N-terminus, and the N-terminus interacts with the CD-loop (C54-K219) (Fig. 6d–h). These interactions stabilize the G-loop in the open state. Moreover, the crystal structures of Kir channels also show that the opening of the G-loop is PIP_2_ independent, for example Kir2.2-apo (PDB code: 3JYC) and Kir2.2-holo (PDB code: 3SPI), have open and closed G-loop, respectively. The counterclockwise rotation of CTD leads to the kinking of the M2 helix and the opening of the G-loop gate. As has been shown for the X-ray structure of Kir3.2 channel^47^, rotation of the CTD facilitates the gating of Kir channels.

Our data show that the opening of the G-loop gate precedes the opening of the inner helix gate (Figs 5a, b and 6a). However, there is still an open question: is the G-loop gate coupled to the inner helix gate? Undoubtedly, more experiments will be needed to answer these questions.

## Additional Information

**How to cite this article**: Li, J. *et al.* Identification of the Conformational transition pathway in PIP_2_ Opening Kir Channels. *Sci. Rep.* **5**, 11289; doi: 10.1038/srep11289 (2015).
